# Transient Global Amnesia and High-Grade Styloidogenic Jugular Vein Stenosis

**DOI:** 10.1001/jamanetworkopen.2026.0065

**Published:** 2026-02-26

**Authors:** Mohamed S. Muneer, Kellen Vo Vu, Siddhant S. Dhawan, Yusef Qazi, Thomas R. Geisbush, Tarik F. Massoud

**Affiliations:** 1 Division of Neuroimaging and Neurointervention, Department of Radiology, Stanford University School of Medicine, Stanford, California; 2 MD Program, Weill Cornell Medicine, New York, New York; 3 Department of Bioengineering, Stanford University Schools of Engineering and Medicine, Stanford, California; 4 allGood, Palo Alto, California

## Abstract

**Importance:**

Pathogenesis of focal hippocampal ischemia in transient global amnesia (TGA) remains unclear but may be of venous origin. Brain magnetic resonance imaging (MRI)–restricted diffusion has considerable limitations as the only current TGA imaging marker, highlighting a critical need for more reliable disease determinants.

**Objective:**

To assess whether the severity of unilateral or bilateral styloidogenic jugular vein compression (SJVC), which can impair intracranial venous drainage, could serve as a novel adjunctive imaging risk factor associated with TGA diagnosis.

**Design, Setting, and Participants:**

This retrospective case-control study of patients with TGA and hippocampal-restricted diffusion and without TGA but with hippocampal-restricted diffusion and healthy controls was conducted from May 2016 to June 2024 in a single large academic medical center.

**Exposure:**

A brain MRI including diffusion-weighted imaging and extended multiplanar volumetric postcontrast sequences revealing the internal jugular veins (IJVs) in the upper neck.

**Main Outcomes and Measures:**

Calculations of cross-sectional areas and the percentage of SJVC stenosis in both IJVs were assessed. The primary outcome was the association of right, left, and combined bilateral SJVC stenosis with TGA status. Secondary measures included IJV baseline size and asymmetry.

**Results:**

There were 84 approximately age-matched and sex-matched patients and healthy controls (mean [SD] age, 66 [11.7] years) included in this study; 44 (52.4%) were female, and 40 (47.6%) were male. All participants had brain MRIs: 28 with clinical TGA and hippocampal-restricted diffusion (TGA+/MRI+), 28 without clinical TGA but with incidental hippocampal-restricted diffusion (TGA−/MRI+), and 28 controls without TGA or restricted diffusion (TGA−/MRI−). Right IJVs were larger in all groups, left IJVs were significantly smaller in TGA+/MRI+ than in controls (mean [SD] axial area, 45.0 [30.0] mm^2^ vs 60.7 [27.5] mm^2^; *P* = .04), and the combined IJV caliber was significantly smaller in TGA+/MRI+ than in controls (mean [SD] axial areas, 126.7 [37.1] mm^2^ vs 150.6 [28.8] mm^2^; *P* = .009). Left SJVC stenosis was not associated with patient cohort, but right mean (SD) SJVC stenosis was significantly greater in TGA+/MRI+ than in TGA−/MRI+ (70.4% [30.7%] vs 56.7% [24.1%]; *P* = .01) and in controls (70.4% [30.7%] vs 51.8% [20.1%]; *P* = .001). Combined mean (SD) stenosis was also greater in TGA+/MRI+ than in controls (67.5% [24.9%] vs 54.6% [16.1%]; *P* = .01). Logistic regression demonstrated that right SJVC stenosis (odds ratio, 1.36 [95% CI, 1.06-1.75]; *P* = .007) and combined stenosis (odds ratio, 1.38 [95% CI, 1.02-1.86]; *P* = .02) were associated with TGA when compared with controls.

**Conclusions and Relevance:**

In this case-control study of patients with TGA vs healthy participants, TGA was associated with high-grade SJVC in the presence of small-caliber IJVs, with an approximately 70% right-sided or bilateral IJV stenosis threshold emerging as a promising imaging covariate for supporting the clinical diagnosis of TGA. These results suggest that MRI assessment of SJVC severity may improve diagnostic accuracy in atypical TGA presentations.

## Key Points

**Question:**

Could styloidogenic jugular vein compression (SJVC) severity represent a novel adjunctive imaging risk factor associated with a transient global amnesia (TGA) diagnosis?

**Findings:**

In this case-control study of 84 patients, right-sided SJVC stenosis (70% vs 52%) and combined right and left stenosis (68% vs 55%) were significantly greater in patients with TGA vs healthy controls.

**Meaning:**

The findings of this study suggest that TGA is associated with high-grade SJVC in patients with small-caliber internal jugular veins, with a right-sided or bilateral stenosis of approximately 70% as a potential risk factor for TGA.

## Teaser

This case-control study assesses whether unilateral or bilateral styloidogenic jugular vein compression severity could serve as a novel adjunctive imaging factor associated with a transient global amnesia diagnosis.

## Introduction

Transient global amnesia (TGA) is an acute, self-limited syndrome marked by sudden anterograde and retrograde amnesia lasting up to 24 hours.^1,2^ It affects 3 to 8 per 100 000 people annually, typically between the ages of 50 and 70 years.^1,2^ The cause remains unclear but is likely multifactorial, with possible mechanisms including vascular ischemia, migraine, epileptic activity, psychogenic factors, and hippocampal dysfunction especially in the cornu ammonis area 1 region.^1^ A further proposed mechanism that has attracted particular debate is venous outflow impairment in the neck, potentially leading to transient intracranial hypertension and secondary hippocampal ischemia.^3,4^

**Figure 1.**
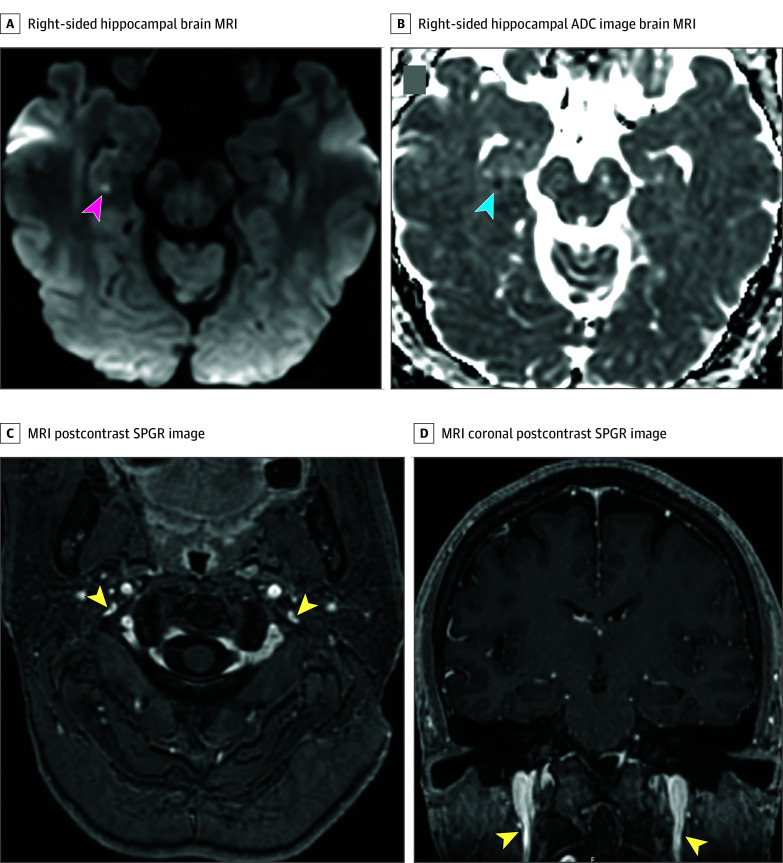
Brain Magnetic Resonance Imaging (MRI) Appearances and the Corresponding Severe Styloidogenic Jugular Venous Compression (SJVC) Stenosis of the Internal Jugular Veins (IJVs) in the Upper Neck of a 61-Year-Old Female With Clinical Transient Global Amnesia (TGA) A, Focal punctate right-sided hippocampal hyperintensity (pink arrow) of axial MRI-diffusion–weighted imaging associated with restricted diffusion. The patient was imaged 24 h after TGA onset. B, The corresponding axial apparent diffusion coefficient (ADC) image shows hypointensity (blue arrow) of the same lesion. C, The MRI axial postcontrast spoiled gradient echo (SPGR) image shows that the patient had severe narrowing of bilateral IJVs (yellow arrows) associated with SJVC. D, A corresponding coronal postcontrast SPGR image shows the same severe SJVC narrowing of bilateral IJVs (yellow arrows).

Diagnosis of TGA is primarily clinical, based on established criteria (Caplan^5^; Hodges and Warlow^6^): acute severe memory impairment lasting 1 to less than 24 hours, being without focal deficits, impaired consciousness, head trauma, or epilepsy. These features distinguish it from stroke, epileptic amnesia, and other mimics. Diagnosis is usually clear in the acute phase, but atypical cases require additional testing.^1^ Magnetic resonance imaging (MRI) is most useful to exclude alternatives and may show punctate hippocampal diffusion-weighted imaging (DWI) lesions (Figure 1A and B),^7^ best detected 24 to 72 hours after onset, and often used as supportive but not definitive imaging markers. However, these MRI lesions have some limitations as TGA markers: (1) they are time dependent, disappearing in 7 to 10 days—DWI lesions undergo pseudonormalization after this period; (2) they appear in only approximately 75% of patients with TGA,^8^ so their absence does not rule out the disease; (3) atypical presentations requiring MRI are common, with 25% of patients with acute amnesia in the Hodges and Warlow cohort not meeting strict criteria^6^; and (4) because recurrence (≤3 episodes) occurs in 3% to 27% of patients,^9^ the lack of more reliable imaging explanatory variables may contribute to the current absence of evidence-based prophylaxis in patients considered for clinical follow-up.^1^

Styloidogenic jugular venous compression (SJVC), in which the internal jugular vein (IJV) is compressed between the C1 transverse process, the styloid process, and the posterior belly of the digastric muscle (Figure 1C and D), is a common anatomical variant.^10^ It is usually asymptomatic but has been associated with idiopathic intracranial hypertension,^11^ raising the possibility that it could contribute to impaired cerebral venous drainage in patients who are susceptible. Han et al^12^ previously reported a correlation between SJVC and TGA; however, their study had notable limitations: (1) SJVC was graded only visually on MR images without quantitative assessment, (2) normal baseline IJV calibers were not measured to calculate accurate stenosis or assess relative IJV dominance, (3) potential cumulative effects of bilateral IJV narrowing were not evaluated, and (4) no logistic regression association modeling or clinical recommendations were proposed.

In this proof-of-concept study, we hypothesized that severe SJVC—particularly when affecting the dominant right IJV or occurring bilaterally—may contribute to impaired intracranial venous outflow sufficient to provoke hippocampal venous ischemia in patients with TGA. Restriction of overall drainage through a single dominant IJV or through both IJVs could create a physiological bottleneck, amplifying venous congestion during transient triggering events. We therefore investigated the potential association between TGA and bilateral SJVC by quantitative analysis of IJV percentage stenosis in patients with hippocampal DWI lesions, aiming to provide a more objective and reproducible assessment than prior reports. A further goal was to better inform clinical diagnostic pathways by finding improved or additional imaging risk factors for TGA, which may be particularly valuable in atypical or diagnostically challenging presentations.

## Methods

This retrospective case-control study was approved by the Human Investigation Committee (institutional review board) of Stanford University School of Medicine. This included a waiver of individual authorization for recruitment under title 45 CFR 164.512, pursuant to information provided in the HIPAA (Health Insurance Portability and Accountability Act) section of the protocol application. Informed consent was not required, as patient information was anonymized, and this study does not include images that may identify patients. This study followed the Strengthening the Reporting of Observational Studies in Epidemiology (STROBE) reporting guideline for case-control studies.

### Patient Selection

We retrospectively screened radiology reports from May 2016 to June 2024 and set aside reports mentioning TGA in adult patients. We manually reviewed these reports and selected patients with TGA and controls for further analysis. We arrived at this analysis group by first identifying patients with clinically confirmed TGA who also had positive MRI findings of hippocampal MRI-DWI restriction (TGA+/MRI+). The MRIs of 5 patients were inadequate for assessing styloidogenic venous compression on the 3-dimensional–spoiled gradient recalled echo (SPGR) images in the TGA+/MRI+ group. A comparison group was then chosen by sequentially reviewing patients without clinical TGA but with MRI findings also depicting hippocampal DWI restriction (TGA−/MRI+); we included these patients who met the approximate age-matching and sex-matching criteria. We then selected a third cohort from patients (negative for brain metastases) of similar size and age and sex matching as controls without TGA or relevant MRI findings (TGA−/MRI−).

### Imaging Parameters and Image Analysis

All brain MRIs were performed on 3-T units (Discovery MR450, Optima MR450, and Signa HDx, GE HealthCare) with standard 8-element head coils and a uniform brain protocol, including DWI and apparent diffusion coefficient sequences, and multiplanar volumetric postcontrast SPGR sequences extending to the upper neck to capture SJVC (echo time, 3 ms; repetition time, 6.8 ms; field of view, 24 cm; 256 × 256 mm matrix; and 0.9 mm slices). A neuroradiologist with 37 years’ experience (T.F.M.) and a trainee with 4 years’ experience (M.S.M.) concurrently and collaboratively analyzed together all anonymized images of the patients and controls on a Sectra PACS (picture archiving and communication system), version IDS7 review workstation. We noted the presence and side of hippocampal-restricted diffusion for patients with TGA and then used click-and-drag electronic calipers in the PACS built-in image tools to obtain all morphometric measurements on the digital postcontrast SPGR images that optimally showed the IJVs and SJVCs. Objective quantitative image analysis was conducted in a semiblinded manner, meaning that datasets were anonymized, but complete blinding to cohort type could not be guaranteed for all study participants. We measured anteroposterior and transverse diameters of each IJV in axial planes and also confirmed in coronal and sagittal views. Any minor dissimilarities (notably, there was an excellent reliability coefficient of >0.90 for first-time agreements) arising between raters (M.S.M. and T.F.M.) as they jointly performed the quantitative objective or unambiguous measurements were discussed and settled by additional consensus agreement.

### Data Collection

We measured diameters of each IJV at the level of styloidogenic compression and 2 centimeters above, confirmed in multiple planes. Cross-sectional areas and stenosis percentages were calculated as: percent stenosis = {1 – [(D_stenosis1_ × D_stenosis2_)/(D_normal1_ × D_normal2_)]} × 100, in which D is the vessel diameter. Stenosis was categorized as nonsevere (<80%) or severe (≥80%). We derived combined bilateral stenosis from summed cross-sectional areas and assessed right and left associations separately.

### Statistical Analysis

We conducted analyses in Python, version 3.12 (Python Software Foundation) and Excel 2021 (Microsoft Corp). We computed descriptive statistics for demographic variables, hippocampal lesion status, and IJV dominance characteristics for all study participants. Kruskal-Wallis tests compared right, left, and combined SJVC stenosis between cohorts (TGA+/MRI+, TGA−/MRI+, and controls). Dunn post hoc tests with Holm-Bonferroni correction addressed pairwise comparisons (significance at 2-sided *P* < .05). To quantify the association between SJVC stenosis and TGA, we performed logistic regression analyses using right SJVC stenosis, left SJVC stenosis, and combined bilateral SJVC stenosis as predictors. TGA status (TGA+/MRI+ vs TGA−/MRI+ or TGA+/MRI+ vs controls) was considered the outcome variable. We reported odds ratios (ORs) with 95% CIs and *P *values from the likelihood ratio tests.

## Results

Among screened radiology reports, 313 mentioning TGA in adult patients were set aside. Of these, 84 patients selected for analysis were approximately age-matched and sex-matched (mean [SD] age, 66 [11.7] years; 44 females [52.4%] and 40 males [47.6%]), had DWI and postcontrast 3-dimensional–SPGR brain MRI sequences, and were divided into 3 groups. After exclusions among the 33 patients first confirmed with TGA who also had positive MRI findings of hippocampal MRI-DWI restriction, the 3 groups included 28 TGA+/MRI+, 28 TGA−/MRI+ (after evaluation of stroke or suspected stroke, epilepsy, or incidentally during assessment of brain metastases), and 28 controls (TGA−/MRI−). Patient age and sex distributions, IJV relative dominance, and sidedness of SJVC for the 3 patient cohorts are shown in Table 1.

**Table 1.** Demographics, Hippocampal Lesion, and IJV Dominance Characteristics for All Study Participants

Characteristics	Participants, No. (%)			
	Total (N = 84)	TGA+/MRI+ (n = 28)	TGA−/MRI+ (n = 28)	TGA−/MRI− (control) (n = 28)
Age, mean (SD), y	66.0 (11.7)	65.2 (10.4)	68.8 (10.9)	64.8 (13.7)
Sex				
Female	44 (52.4)	15 (53.6)	14 (50.0)	15 (53.6)
Male	40 (47.6)	13 (46.4)	14 (50.0)	13 (46.4)
Hippocampal DWI punctate restriction, side				
Right	NA	9 (32.1)	13 (46.4)	NA
Left	NA	17 (60.7)	14 (50.0)	NA
Bilateral	NA	2 (7.1)	1 (3.6)	NA
IJV relative dominance, side				
Right	60 (71.4)	23 (82.1)	16 (57.1)	21 (75.0)
Left	24 (28.6)	5 (17.9)	12 (42.9)	7 (25.0)
Right SJVC stenosis				
Nonsevere	64 (76.2)	14 (50.0)	24 (85.7)	26 (92.9)
Severe	20 (23.8)	14 (50.0)	4 (14.3)	2 (7.1)
Mean (SD) [95% CI], %	59.6 (26.2) [53.9-65.3]	70.4 (30.7) [58.5-82.3]	56.7 (24.1) [47.4-66.0]	51.8 (20.1) [44.0-59.6]
Left SJVC stenosis				
Nonsevere	63 (75.0)	20 (71.4)	18 (64.3)	25 (89.3)
Severe	21 (25.0)	8 (28.6)	10 (35.7)	3 (10.7)
Mean (SD) [95% CI], %	61.2 (24.3) [55.9-66.5]	62.1 (26.9) [51.6-72.6]	64.4 (23.8) [55.2-73.6]	57.1 (22.1) [48.5-65.7]
Combined right and left SJVC stenosis, mean (SD) [95% CI], %	NA	67.5 (24.9) [57.8-77.2]	NA	54.6 (16.1) [48.3-60.9]

Abbreviations: DWI, diffusion-weighted imaging; IJV, internal jugular vein; MRI, magnetic resonance imaging; NA, not applicable; SJVC, styloidogenic jugular venous compression; TGA, transient global amnesia.

**Figure 2.**
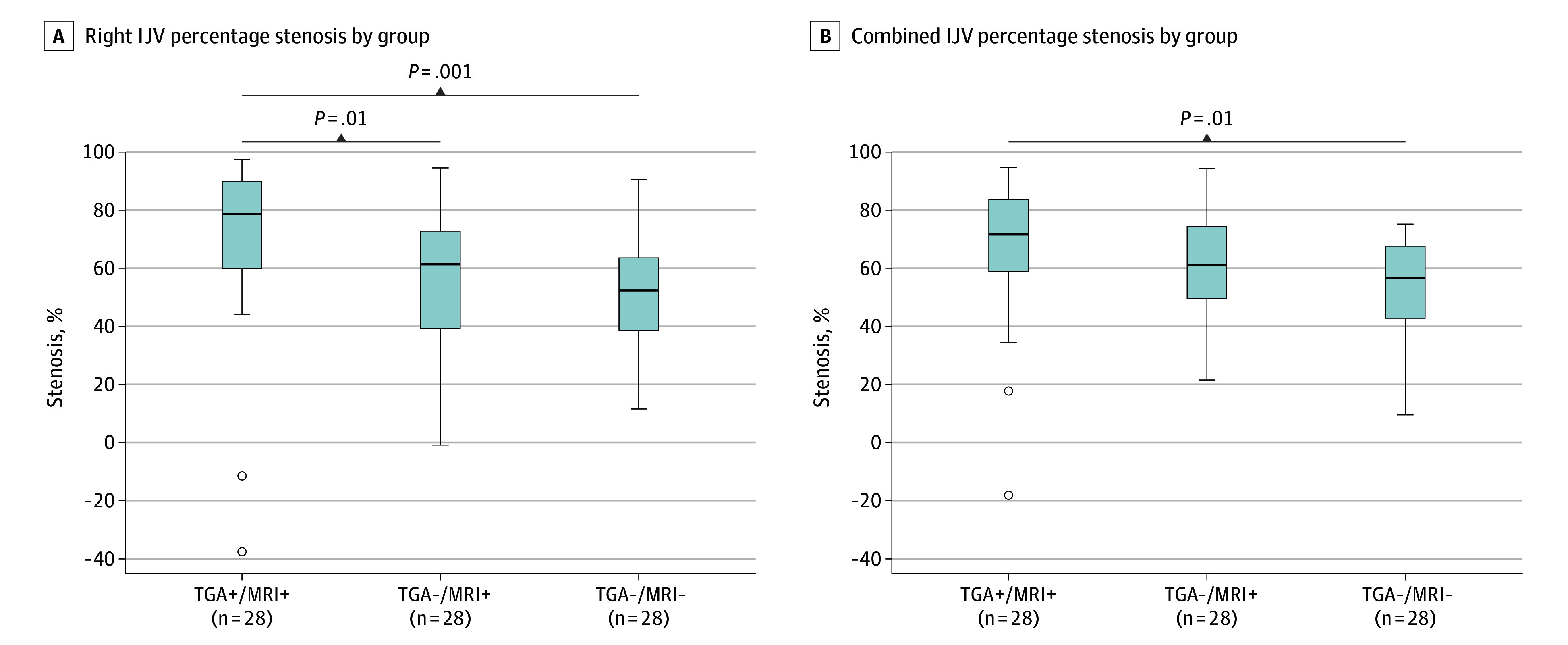
Box and Whisker Plots of Statistical Associations of Right and Combined Styloidogenic Jugular Venous Compression Stenosis by Patient Cohort Category Right (A) and combined (B) internal jugular vein (IJV) mean percentage stenosis is shown in patients in the transient global amnesia (TGA)+/magnetic resonance imaging (MRI)+ and the TGA−/MRI+ groups and in the TGA−/MRI− (control) group. The top and bottom of the boxes indicate IQRs (Q1 to Q3; the middle 50% of the data). The horizontal lines inside the boxes indicate medians; whiskers, extent of the smallest and largest data points within 1.5 × IQR; circles, outlier data more extreme than the whiskers (values beyond 1.5 × IQR).

The means and SDs for all measured parameters are shown in Table 2. We found no associations between left SJVC percentage stenosis and patient cohort category. Hence, left SJVC stenosis alone was disregarded in further analyses. Conversely, right SJVC stenosis was significantly associated with patient cohort category, with mean (SD) stenosis of 70.4% (30.7%) in TGA+/MRI+, 56.7% (24.1%) in TGA−/MRI+, and 51.8% (20.1%) in controls (Kruskal-Wallis, *P* = .001) (Figure 2A). We found no statistically significant difference between TGA−/MRI+ and controls (mean [SD] stenosis, 56.7% [24.1%] vs 51.8% [20.1%]; *P* = .37). We also observed no association between right SJVC stenosis and age, sex, IJV dominance, or side of hippocampal DWI lesion.

**Table 2.** IJV Morphometrics in All Study Participants

Measurements, mm	Participants, mean (SD)			
	Total (N = 84)	TGA+/MRI+ (n = 28)	TGA−/MRI+ (n = 28)	TGA−/MRI− (control) (n = 28)
Right AP at styloidogenic compression	3.7 (1.9)	3.5 (2.5)	3.4 (1.8)	4.0 (1.3)
Right TV at styloidogenic compression	8.3 (3.5)	6.4 (3.9)	8.7 (3.0)	10.0 (2.4)
Right AP cephalad to the compression	8.3 (1.9)	8.3 (1.7)	8.1 (2.3)	8.4 (1.7)
Right TV cephalad to the compression	9.7 (2.3)	9.7 (2.0)	9.1 (2.6)	10.4 (2.2)
Left AP at styloidogenic compression	2.9 (1.4)	2.8 (1.3)	2.7 (1.3)	3.3 (1.4)
Left TV at styloidogenic compression	6.5 (3.1)	5.5 (3.5)	6.9 (3.1)	7.2 (2.5)
Left AP cephalad to the compression	6.5 (1.8)	6.1 (1.9)	6.7 (2.0)	6.8 (1.5)
Left TV cephalad to the compression	8.1 (2.4)	6.9 (2.0)	8.6 (2.6)	8.7 (2.3)

Abbreviations: AP, anteroposterior; IJV, internal jugular vein; MRI, magnetic resonance imaging; TGA, transient global amnesia; TV, transverse.

To investigate the origin of the significant difference in right SJVC between patients with TGA and controls, we focused on 2 additional sets of statistical analyses: (1) the association of right vs left SJVC stenosis for these 2 cohorts and (2) whether the right plus left (combined) SJVCs’ stenosis was similarly associated with patients with TGA rather than controls.

As SJVC percentage stenosis is defined by both baseline size of the IJVs (Table 2) and the degree of additional luminal reduction, we further analyzed baseline sizes of IJVs in patients with TGA and controls. Using within-group comparisons (paired *t* tests), we found that the right IJV was significantly larger than the left IJV in both patients with TGA (mean [SD] right axial area, 81.7 [25.5] mm^2^ vs left axial area, 45.0 [30.0] mm^2^; *P* < .001) and controls (mean [SD] right axial area, 89.8 [31.4] mm^2^ vs left axial area, 60.7 [27.5] mm^2^; *P* = .006), but an independent *t* test showed that this asymmetry was not significantly different between the 2 cohorts. For left (but not right) IJVs, the size was significantly larger in controls than in patients with TGA (mean [SD] axial areas, 60.7 [27.5] mm^2^ vs 45.0 [30.0] mm^2^; *P* = .04). Notably, combined sizes of right IJVs plus left IJVs (Table 2) were significantly smaller in patients with TGA compared with controls (mean [SD] axial areas, 126.7 [37.1] mm^2^ vs 150.6 [28.8] mm^2^; *P* = .009).

Next, we tested whether the novel metric of combined right and left SJVC stenosis relative to baseline caliber of bilateral IJVs would also be associated with patients with TGA rather than with controls. The degree of combined SJVC stenosis was associated with the patient cohort category, with mean (SD) stenosis of 67.5% (24.9%) in TGA+/MRI+, 60.2% (19.9%) in TGA−/MRI+, and 54.6% (16.1%) in controls (Kruskal-Wallis, *P* = .01) (Figure 2B). We found no statistically significant difference in combined SJVC stenosis between TGA+/MRI+ and TGA−/MRI+ (mean [SD] combined stenosis, 67.5% [24.9%] and 60.2% [19.9%]; *P* = .14) or TGA−/MRI+ and controls (mean [SD] combined stenosis, 60.2% [19.9%] and 54.6% [16.1%]; *P* = .24) groups. There was also no association between combined SJVC stenosis and age, sex, IJV dominance, or side of hippocampal DWI lesion.

In terms of proportional associations of right or left percentage stenosis with the combined bilateral SJVC percentage stenosis, the right mean (SD) percentage stenosis was significantly more than the left one (61.6% [20.6%] vs 38.4% [20.6%]; *P* = .006) for controls, but in patients with TGA, there was no significant difference between right and left mean (SD) percentage stenosis (56.0% [22.4%] vs 44.0% [22.4%]; *P* = .16).

We compared patients in the TGA+/MRI+ group with those in the TGA−/MRI+ group and controls separately using logistic regression analysis. Between TGA+/MRI+ and TGA-/MRI+, there were no associations among the right stenosis (OR, 1.22 [95% CI, 0.98-1.51]; *P* = .06), combined stenosis (OR, 1.16 [95% CI, 0.91-1.49]; *P* = .22), or the left stenosis (OR, 0.96 [95% CI, 0.78-1.19]; *P* = .07) . When comparing patients in the TGA+/MRI+ group with controls, there was an association with TGA status for both right stenosis (OR, 1.36 [95% CI, 1.06-1.75]; *P* = .007) and combined bilateral SJVC stenosis (OR, 1.38 [95% CI, 1.02-1.86]; *P* = .02) but not left stenosis (OR, 1.09 [95% CI, 0.87-1.36]; *P* = .44).

## Discussion

Given the as-yet unclear role of venous congestion in TGA pathogenesis,^13^ in this case-control study, we conjectured that prominent SJVC stenosis may contribute to this disease. Indeed, IJVs represent the dominant outflow pathway for cerebral venous blood in most individuals (72%), but alternative extrajugular routes usually compensate if IJVs are obstructed.^14^ However, in patients with TGA with high-grade right or bilateral IJV stenosis, especially with small-caliber IJVs (as we observed), it is plausible that venous return may be critically impaired during Valsalva-like maneuvers. Such triggers—physical exertion, sexual activity, or swimming—may precede TGA in up to 44% of presentations,^15^ possibly supporting a venous mechanism in patients who are susceptible.^3^

Although recurrence rates are low for TGA, the transient but dramatic memory loss can cause significant distress to patients and families. However, the risk of intervention is not warranted given the benign nature of TGA, even when recurrent and even with the knowledge that there are possible invasive treatments for severe SJVC associated with other neurological conditions.^16^ Importantly, the presence of severe SJVC in some patients with TGA may inform an evidence-based prophylaxis and recommendation program during clinical follow-up.

### Strengths and Limitations

This study has strengths. Our proof-of-concept study benefits from a large, single-center representative population with TGA, 2 non-TGA control cohorts, and detailed morphometric and statistical analyses of IJV features.

The study also has limitations. Its limitations include potential selection bias and a retrospective design that supports hypothesis generation but not causal proof. Associations in this study may still represent type I error, as statistical significance at *P* < .05 does not eliminate the possibility of false positives. Conversely, the nonsignificant results that we obtained may reflect limited power and thus carry risk of type II error, particularly for smaller effect sizes. Future replication in larger TGA cohorts prospectively accrued from multiple centers will be needed to support the robustness of our findings.

## Conclusions

In this case-control study of patients with TGA vs healthy participants, we found that TGA was associated with high-grade SJVC in patients with small-caliber IJVs. Logistic regression analysis showed that an approximately 70% right or combined right and left IJV stenosis represented a meaningful imaging feature associated with TGA and may serve as a supportive finding in appropriate clinical contexts. Particularly in atypical presentations or when hippocampal-restricted diffusion is questionable or absent, we recommend incorporating evaluation of right or bilateral SJVC severity into routine MRI assessment of patients with TGA.

## Notes

Supplement.
Data Sharing Statement
