# Supplementary material for: Transient Global Amnesia and High-Grade Styloidogenic Jugular Vein Stenosis
Source: JAMA Netw Open. 2026 Feb 26;9(2):e260065. doi: 10.1001/jamanetworkopen.2026.0065 (PMC12947034; doi:10.1001/jamanetworkopen.2026.0065)
Supplement: Supplement. — Data Sharing Statement [file jamanetwopen-e260065-s001.pdf]

Data Sharing Statement

Muneer. Transient Global Amnesia and High-Grade Styloidogenic Jugular Vein Stenosis. *JAMA Netw Open*. Published February 26, 2026. doi:10.1001/jamanetworkopen.2026.0065

Data
Data available: Yes
Data types: Deidentified participant data
How to access data: upon reasonable request from corresponding author at tmassoud@stanford.edu
When available: With publication

Supporting Documents
Document types: None

Additional Information
Who can access the data: researchers whose proposed use of the data has been approved
Types of analyses: for research purposes
Mechanisms of data availability: with investigator support
Any additional restrictions: no additional information
